# Intestinal *Bacteroides* sp. Imbalance Associated With the Occurrence of Childhood Undernutrition in China

**DOI:** 10.3389/fmicb.2019.02635

**Published:** 2019-11-29

**Authors:** Dongfang Li, Yinhu Li, Wenkui Dai, Huihui Wang, Chuangzhao Qiu, Su Feng, Qian Zhou, Wenjian Wang, Xin Feng, Kaihu Yao, Yanhong Liu, Yonghong Yang, Zhenyu Yang, Ximing Xu, Shuaicheng Li, Jurong Wei, Ke Zhou

**Affiliations:** ^1^Wuhan National Laboratory for Optoelectronics, Huazhong University of Science and Technology, Wuhan, China; ^2^Department of Microbial Research, WeHealthGene Institute, Shenzhen, China; ^3^Department of Computer Science, College of Science and Engineering, City University of Hong Kong, Kowloon Tong, Hong Kong; ^4^Department of Clinical Nutrition, Shenzhen Children’s Hospital, Shenzhen, China; ^5^Institute of Statistics, Nankai University, Tianjin, China; ^6^Department of Respiratory Diseases, Shenzhen Children’s Hospital, Shenzhen, China; ^7^Department of Respiratory Diseases, Beijing Children’s Hospital, Beijing, China

**Keywords:** childhood undernutrition, gut microbiome markers, *Bacteroides*, iron transporter, nutritional indicators

## Abstract

Undernutrition (UN) is a worldwide concern affecting morbidity and mortality among children, but the safety and long-term efficacy of its current treatments remain controversial. Recent evidence showing the roles of the gut microbiome (GM) in nutrient absorption indicates its usefulness in alternative interventions to treat UN safely with sustainable amelioration. To enhance our understanding of the GM and childhood undernutrition, we deep sequenced the gut metagenomes of 65 children with moderate or severe undernutrition (UN group) and 61 healthy children (HC group) to identify associated taxa and genes using a two-stage validation scheme. At stage I, 54 UN patients and 51 healthy children were enrolled for the discovery of GM markers in UN children. The accuracy of the markers was then tested in an additional 11 UN patients and 10 healthy children at stage II. Compared to the HC group, the UN group had lower richness in microbial genes (*P* = 0.005, FDR = 0.005) and species (*P* = 0.002, FDR = 0.002). The distributions of bacterial genes enable the identification of 16 gene markers with which to discriminate UN patients with high accuracy [averaged areas under the receiver operating curve (AUC) = 0.87], including three *Bacteroides uniformis* genes that are responsible for the synthesis of iron transporters. We also identified four species markers that enable the UN patients to be confidently discriminated from the HC children (averaged AUC = 0.91), namely *Bacteroides ovatus*, *Bacteroides uniformis*, *Bacteroides uniformis*, and *Bacteroides vulgatus*. In addition, metabolic comparison showed significantly decreased isobutyric acid (*P* = 0.005, FDR = 0.017) and increased isovaleric acid (*P* = 0.006, FDR = 0.017) in UN patients. We also identified notable correlations between microbial species and short-chain fatty acids (SCFAs) and several nutritional indicators, including acetic acid and iron (*r* = 0.436, *P* = 0.029), butyric acid and iron (*r* = 0.422, *P* = 0.036), butyric acid and lymphocyte (*r* = −0.309, *P* = 0.011), and acetic acid and total protein (*r* = −0.303, *P* = 0.043). Taken together, the distinct features of gut microbiota in UN patients highlight the taxonomic and functional shift during the development of UN and provide a solid theoretical basis for intervention in childhood undernutrition through gut microbes.

## Introduction

Childhood undernutrition is caused by an imbalance between energy intake and energy expenditure (de Clercq et al., 2016), and the weight-for-age Z score (WAZ) as defined by the World Health Organization [WHO], 2017 is normally applied for diagnosis of the disease (de Onis et al., 2004; World Health Organization [WHO], 2017). According to an investigation in 2014, approximately 159 million children suffered from undernutrition worldwide (UNICEF et al., 2015), contributing to over 40% of deaths in children under 5 years of age (Bhutta et al., 2013). Childhood undernutrition is not merely associated with growth stunting: it also poses persistent adverse impacts on hosts, such as immune dysfunctions, neurocognitive deficits, and endocrine system disorders (Prendergast and Humphrey, 2014). Although current therapeutic strategies [such as supplying adequate nutrients (Lenters et al., 2013) and antibiotic administration (Trehan et al., 2016)] could improve growth in most undernourished children, some patients exhibit little clinical amelioration (Gough et al., 2014), and the safety and long-term efficacy of these treatments remains controversial (Gough et al., 2014; Trehan et al., 2016). With an increasing number of studies on the gut microbiome (GM) (Smith et al., 2013; Subramanian et al., 2014; Blanton et al., 2016b), more and more researchers are focusing on the sustainable repair ability of the GM and its roles in childhood undernutrition (Blanton et al., 2016a; Du Toit, 2016).

Previous studies have indicated the intimate associations between the GM and childhood undernutrition (Smith et al., 2013; Subramanian et al., 2014; Blanton et al., 2016b; Wagner et al., 2016). A large cohort study on Bangladesh children suggested the persistent immature GM in undernutrition (UN) children as compared with healthy (HC) children (Subramanian et al., 2014), and the growth deficit was observed in mouse models when transferred with the fecal from UN patients (Blanton et al., 2016b; Charbonneau et al., 2016). Nevertheless, the mechanisms by which the GM regulates the energy homeostatic in host were also clarified. By activating insulin like growth factor 1 (IGF-1), *Lactobacillus plantarum* would improve energy harvest, body growth, and the somatropic axis in host (Yan et al., 2016). On the other hand, GM secreted acetate could repress fat synthesis and increase energy expenditure (Sahuri-Arisoylu et al., 2016). Based on the above evidences, we know that the GM (Blanton et al., 2016a, b) and its metabolites (Cani et al., 2019; Serino, 2019) contributed to the occurrence of undernutrition.

More recently, the casual relationships between the GM and nutritional status were confirmed (Sanna et al., 2019). Through bidirectional Mendelian randomization experiments, Serena Sanna et al. (2019) have discovered that GM and fecal species and short-chain fatty acids (SCFAs) were associated with the insulin response and the risks of obesity in hosts. Furthermore, the representative bacteria that related with the occurrence of undernutrition were reported on Malawi and Bangladeshi children (Subramanian et al., 2014; Blanton et al., 2016b). However, since the GM was affected by various factors, such as genetic (Deschasaux et al., 2018), life styles (Chewapreecha, 2014; Zmora et al., 2019), and the exposure of antibiotics and probiotics (Ruiz et al., 2017), distinct GM characters exist among UN patients from different populations. In this study, we proposed to explore the characters of the GM in Chinese UN patients, and detect the relationships between the GM and childhood undernutrition.

To explore the features of the GM in UN children, we recruited 65 children with moderate to severe undernutrition, and 61 healthy children as controls. In addition to characterizing the GM in UN children, we also aimed to elucidate: (i) GM biomarkers for childhood undernutrition; (ii) the associations between GM components and SCFA concentrations; and (iii) the correlations between SCFAs and nutritional indexes.

## Materials and Methods

### Participant Recruitment

**FIGURE 1:**
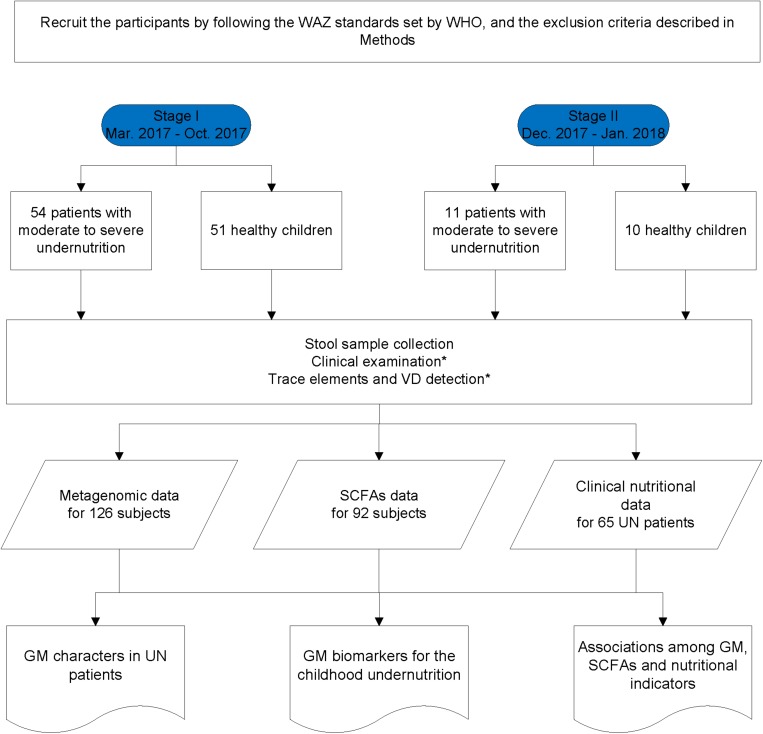
Flow diagram of participant selection and data acquisition. ^∗^Means the data was only obtained in the UN patients.

The UN patients recruited from the Department of Clinical Nutrition, Shenzhen Children’s Hospital satisfied the following criteria: (i) younger than 3 years of age; (ii) patients with moderate or severe undernutrition were selected when their WAZ was two standard deviations below the WHO reference value (de Onis et al., 2004; World Health Organization [WHO], 2017). HC children were selected from those who passed the physical examinations of Shenzhen Children’s Hospital and met the following standards: (i) younger than 3 years of age and (ii) no diarrhea in the last 2 weeks. In addition, the participants were excluded from the study if they: (i) had been exposed to an antibiotic, probiotic, or proton pump inhibitor 1 month prior to fecal sample collection; (ii) had a known allergic history (e.g., food allergy, AD, asthma); (iii) had a known hereditary disease (e.g., thalassemia, hereditary deafness, phenylketonuria); (iv) suffered from a known metabolic or autoimmune disease (e.g., obesity, diabetes, rheumatoid arthritis); and (v) had parasitic eggs in their stool. Moreover, some UN patients suffered from respiratory infections, such as bronchitis and pneumonia; the infection information is provided in the Supplementary File S1. In this study, we carried out a two-stage case-control strategy on collected fecal samples. Using such a methodology, we could test the discoveries with different cohorts, detect the batch effect, and avoid the overfitting problem during the construction of models. Following the WAZ standards set by the WHO (detailed in the Methods), 54 UN patients and 51 HC children were recruited from the Department of Clinical Nutrition, Shenzhen Children’s Hospital at stage I between March 2017 and October 2017 (Figure 1 and Table 1). An additional 11 UN patients and 10 HC children were enrolled at stage II between December 2017 and January 2018 (Figure 1, Table 1, and Supplementary File S1).

**TABLE 1:** Demographic and physical features of the 126 study subjects.

	**Stage I**			**Stage II**			
			
	**UN group (*n* = 54)**	**HC group (*n* = 51)**	***P*-value**	**UN group (*n* = 11)**	**HC group (*n* = 10)**	***P*-value**	**Method**
Gender (male)	22	26	0.392	5	5	1	χ^2^ test
Age (month)	13.5 ± 9.5	18.8 ± 9.7	0.005	15.2 ± 9.3	20.3 ± 13.7	0.335	*t*-test
Delivery pattern			0.034			0.561	χ^2^ test
Cesarean	24	21		6	5		
Vaginally	30	24		5	4		
ND^∗^	0	6		0	1		
Feeding pattern			0.253			0.581	χ^2^ test
Breastfeeding	29	22		7	5		
Formula feeding	6	3		1	0		
Mixed	11	19		2	4		
ND^∗^	8	7		1	1		
Weight to age Z score (%)	[−7.49, −2.01]	[−1.56, 4.83]	<0.001	[−5.6, −2.05]	[−1.25, 1.30]	<0.001	Wilcoxon rank- sum test

*ND^∗^: not detected.*

### Sample Collection

During the physical examination, fresh stools were collected from the participants by using sample swabs (iClean, Huachenyang (Shenzhen) Technology Co., Ltd., China) and stored in sterilized tubes (62-558-201, SARSTEDT AG & Co. KG, Germany). The fecal samples were then transferred to a −80°C refrigerator for long-term storage within 30 min. Meanwhile, blood samples were collected from the UN patients (Figure 1). The concentrations of hemoglobin, albumin, total protein, white cell, and lymphocyte were detected by using a blood auto-analyzer (Beckman Coulter AU5800, Brea, CA, United States) as soon as each blood sample was collected. We also examined the trace elements in the serum of the UN patients (SPECTRO ICP-OES analyzer, SPECTRO, Germany), including iron (Fe), Zinc (Zn), and calcium (Ca) (Calder and Jackson, 2000). The concentrations of vitamin D (VD) in the UN patients were analyzed by using a LIAISON^®^ XL automatic chemiluminescence analyzer (DiaSorin, Centralino, Italy).

### DNA Extraction, Library Construction, and Sequencing

DNA was extracted from stools using the QIAamp DNA Stool Mini Kit (Qiagen, Hilden, Germany) according to manufacturer’s protocols. After quality checking (Qubit, Thermo Fisher Scientific, Singapore), the isolated DNA was prepared for library construction (TruSeq DNA kit, Illumina, San Diego, CA, United States) with an insert size of 350 bases. The qualified libraries were then paired-end sequenced as 150 (nt) reads using the HiSeq platform (Illumina, San Diego, CA, United States).

### SCFA Examination

A 1 g sample of the stool of each participant was placed in a tube (Eppendorf Tubes^®^ 3810X, Eppendorf-China, Shanghai, China) and mixed with 1 ml extraction liquid (methanol: acetonitrile: water = 2:2:1) and 1 ml ultra-pure water (D24 UV, Merck Millipore, Darmstadt, Germany). Then the mixture was ultrasound-treated for 30 min (PS-60AL, Shenzhen Leidebang Electronics Co., Ltd., Shenzhen, China) and centrifuged at 5000 rpm for 5 min at 4°C (Heraeus Fresco 17, Thermo Fisher Scientific, Shanghai, China). The resulting supernatant was transferred to a new tube and added to 1 ml of dodecane and 1 ml of 3% phosphate (Shanghai Hengbai Biological Technology Co., Ltd., Shanghai, China). The gas chromatography analyses were performed on the isolated metabolites (Instant Connect Electron Flame Ionization Detector for TRACE^TM^ 1300 GC Series, Thermo Fisher Scientific, Shanghai, China). During the examination, the injection volume, airflow rate, hydrogen flow rate, and carrier gas flow rate were 0.5 μl, 300, 30, and 15 ml/min, respectively. Finally, the concentrations of SCFAs in each sample were calculated based on their standard curves and peak areas (Supplementary File S2).

### Sequencing Data Filtration and Host Sequence Removal

To obtain high-quality data, the raw reads were filtered using a self-programmed script when they contained more than 10 low-quality (<Q20) bases or 15 bases of adapter sequences (Guo et al., 2018). The filtered reads were then aligned to the human genome (hg19) with BWA (version 0.7.13, −*t* = 10) (Li and Durbin, 2010). Using a self-programmed script, the mapped reads (bwa format) were removed, and clean metagenomic data were obtained for further analysis. The metagenomic data without the host genomic sequence have been posted to the NCBI Sequence Read Archive (SRA) Database under the accession number PRJNA543967.

### Bacterial Gene Annotation and Taxonomic Assignment

The processed data were aligned to the updated Integrated Gene Catalog (updated IGC) (Xie et al., 2016) (using BWA, −*t* = 10), and the number of mapped reads was counted for each gene in each sample (Supplementary File S3). The relative abundances of the genes were calculated based on the number of mapped reads (Xie et al., 2016). For example, for a given gene “a,” its relative abundance [Ab(a)relative] can be calculated using the formula below:

A⁢b⁢(a)=U/L

A⁢b⁢(a)⁢r⁢e⁢l⁢a⁢t⁢i⁢v⁢e=A⁢b⁢(a)×100/A⁢b⁢(a⁢l⁢l)

In this formula, Ab(a) is the abundance of gene “a,” while parameters U and L stand for the number of mapped reads for gene “a” and the length of gene “a,” respectively. Ab(all) is the number of all aligned reads in the sample. If the abundances equal 0 in more than 70% of the sample, the gene will be removed from further study.

The species profile was determined using MetaPhlAn2 (version 2.6) (Segata et al., 2012; Truong et al., 2015). By aligning to the specific marker genes identified from reference genomes, the processed data from each sample were used to estimate the relative abundances of the known microbial species, and the species profile was acquired by combining the species abundances from all samples (Supplementary File S4).

### PERMANOVA Analysis

The impact of physical indices (e.g., gender, age, delivery pattern, and feeding pattern) on GM distributions was assessed using Permutational Multivariate Analysis of Variance (PERMANOVA) with 999 permutations and Euclidean distances (package “vegan” in R) (Supplementary File S5; Tang et al., 2016).

### Biomarker Identification and Random Forest Model Construction

The representative gene biomarkers were obtained by removing the genes absent in more than 70% of the samples; this left 191,470 genes. Owing to the high-dimensional features of the genes, a Wilcoxon rank-sum test was first conducted between the UN group and the HC group, and 18 differentially abundant genes were significantly identified (*P* < 0.01 and FDR < 0.01). Then, a random forest model was constructed between the UN group and the HC group from stage I with fivefold cross-validation, and the candidate gene markers were selected based on the Gini values and optimal variation numbers (using the R package “random-Forest”; Liaw and Wiener, 2002). The samples collected at stage II were then employed to evaluate the accuracy of the gene markers and the model using area under the curve (AUC) values with five repeats, and the results were represented as receiver operating characteristic (ROC) curves (Linden, 2006).

Similar to the discovery procedure for gene markers, species were removed when absent in more than 70% of the samples, and 72 species remained for the species marker identification. With 105 samples from stage I, four optimal species were identified for the discrimination of UN patients. The accuracy of the species markers was then estimated in samples at stage II using AUC values with five repeats.

### Statistical Analysis

**FIGURE 2:**
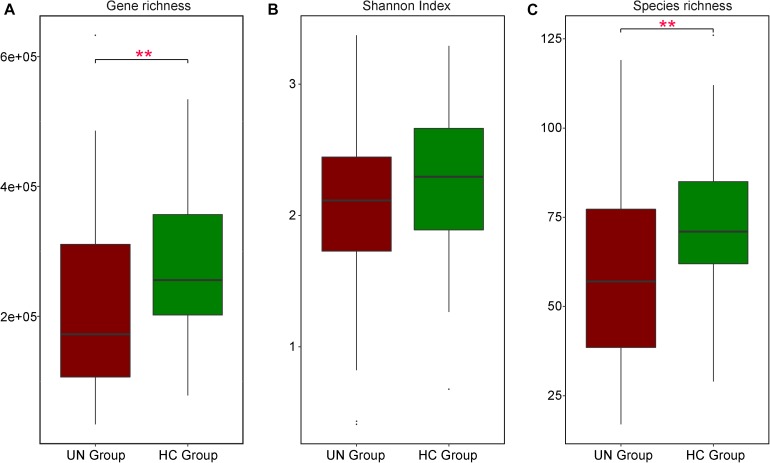
Differences in GM features between UN and HC children. Comparisons on gene richness **(A)**, Shannon index **(B)**, and species richness **(C)** were executed between 54 UN patients and 51 HC children; their values in these two groups are represented by red and green boxes respectively. Significant differences are indicated with ^∗∗^*P* < 0.01.

Based on species profiling of the samples, alpha diversity was assessed with the Shannon index, utilizing the “vegan” package in R (version 3.4.0) (Jari Oksanen et al., 2018). Wilcoxon rank-sum testing was applied to explore differentially enriched bacteria between the UN and the HC groups and was also used to examine the differences in gene richness (Figure 2A), Shannon index (Figure 2B), and species richness (Figure 2C) between these two groups. Spearman correlation analysis was executed among GM species, SCFAs, and clinical nutritional indexes based on their relative abundances or concentrations (using “cor” in R). Statistical results from Wilcoxon rank-sum tests and Spearman correlation analysis were adjusted with the Benjamini and Hochberg method (FDR < 0.05) (Grant et al., 2005) using “p.adjust” in R. ROC curves were plotted with the “pROC” package in R.

## Results

### Characteristics of the Samples

In this study, a two-stage case-control strategy on GM was carried out for the identification of biomarkers in UN children (Qin et al., 2012). At the first stage, 54 children with moderate to severe undernutrition (UN group) and 51 healthy children (HC group) were enrolled for fecal collection (Table 1 and Supplementary File S1). The GM biomarkers discovered at stage I were validated by enrolling an additional 11 UN children and 10 HC children at stage II (Table 1 and Supplementary File S1). With the NGS and data filtration described in the Methods, a total of 768 gigabases (Gb) of data was obtained for the 126 participants. After the removal of host sequences, we obtained 666.88 Gb of metagenomic data for all samples; each sample provided 5.29 ± 1.02 Gb (mean ± SD) high-quality data on average (Supplementary File S3). The reads were then aligned to the updated Integrated Gene Catalog (updated IGC) with a mapping ratio of 93.78 ± 6.59%, and a total of 3,315,701 genes were obtained (Supplementary File S3).

### Distinct GM Characteristics in Undernourished Children

For samples at stage I, the UN group exhibited significantly lower microbial gene richness as compared to the HC group: the microbial gene numbers were 216,429 ± 142,555 and 278,906 ± 112,794 in the UN and HC groups, respectively (*P* = 0.005, FDR = 0.005, Figure 2A). Based on the taxonomic profile, we detected 478 species for all samples and found that the UN group contained significantly fewer annotated species than the HC group (*P* = 0.002, FDR = 0.002, Figure 2C). The numbers of species were 59.72 ± 26.52 and 73.27 ± 18.30 in the UN and HC groups, respectively (Supplementary File S4), and age had an impact on GM difference between the groups (*P* = 0.001, PERMANOVA analysis, Supplementary File S5). Moreover, two age-discriminatory species (*Faecalibacterium prausnitzii* and *Lachnospiraceae bacterium* 5 1 63FAA) were discovered, and their relative abundances increased with increasing age (Supplementary File S6). At baseline, the UN group exhibited 19 differentially abundant GM species as compared with the HC group: *Rothia mucilaginosa* (*P* = 0.002, FDR = 0.007) significantly enriched in the undernourished patients and the others obviously decreased. Among the 18 species decreased in the UN group, nine of them belonged to *Bacteroides*, and the rest were from *Parabacteroides*, *Anaerostipes*, *Prevotella*, *Lachnospiraceae*, *Bifidobacterium*, *Faecalibacterium*, *Eubacterium*, and *Roseburia* (detailed in Supplementary File S7).

### Microbial Gene Biomarkers Discriminating Undernourished Children

**FIGURE 3:**
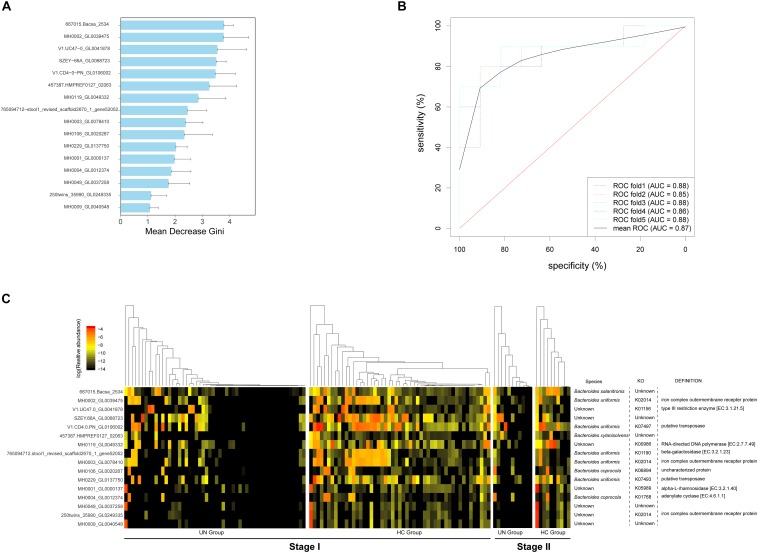
Bacterial gene markers and their validation accuracy for the discrimination of UN patients. **(A)** Following the optimal variation numbers indicated by Random Forest classifiers, 16 gene biomarkers with Gini values applied to indicate their contributions to the discrimination between the UN group and the HC group classification, were selected at stage I. **(B)** These gene markers were tested in the samples from stage II, and their AUC values were calculated. The ROC curves were drawn with five repeats in different colors. **(C)** The log_10_ relative abundances of the gene markers were detected for all samples; the species, KEGG pathways, and functions corresponding to the biomarkers are also exhibited.

We identified 16 microbial gene markers that discriminate the undernourished patients from healthy children with a Random Forest classifier and Wilcoxon rank-sum test on 105 metagenomic samples at stage I (Figure 3A). Besides the un-annotated genes (7 of 16), the other biomarkers were from *Bacteroides*, including *Bacteroides uniformis* (5 of 16), *Bacteroides coprocola* (2 of 16), *Bacteroides salanitronis* (1 of 16), and *Bacteroides xylanisolvens* (1 of 16) (Figure 3C). With the functional annotation results, we discovered that three of the biomarkers participated in the synthesis of “iron complex outer membrane receptor protein” (KO2014, Figure 3C), and their were abundances markedly lower in the UN group than in the HC group (Supplementary File S8). In addition, gene biomarkers were also related with the synthesis of “putative transposase” (2 of 16), “type III restriction enzyme” (1 of 16), RNA-directed DNA polymerase (1 of 16), etc. (Figure 3C). To assess the robustness of the 16 microbial gene markers, we evaluated these biomarkers in 21 samples at stage II. Notably, with high accuracy (mean AUC = 0.87, Figure 3B) markers, the undernourished children can be screened from healthy children.

### Species Biomarkers Selected for Screening for Undernourished Children

**FIGURE 4:**
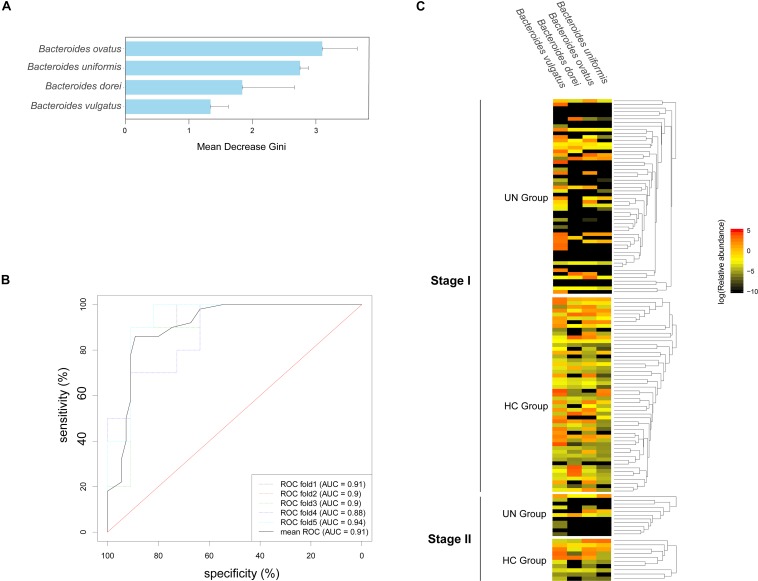
Species markers and their accuracy in differentiating the UN group from the HC group. **(A)** Four species were identified for the optimal classification of the HC and UN children at stage I; the Gini values are plotted. **(B)** The accuracy of the species markers was evaluated by using the samples from stage II, and ROC curves were drawn with five repeats in different colors along with AUC values. **(C)** The relative abundances of the biomarkers in all samples are shown with a heatmap.

The 72 bacterial species that existed in over 30% of the total samples at stage I were selected (Supplementary File S4). Based on their relative abundances and the Random Forest model, four species were identified as biomarkers for the discrimination of undernourished children, namely *Bacteroides ovatus*, *Bacteroides uniformis*, *Bacteroides uniformis*, and *Bacteroides vulgatus* (Figure 4A). Moreover, high accuracy was detected when the biomarkers were applied for the validation samples at stage II (mean AUC = 0.91, Figure 4B). Interestingly, these four biomarkers were from *Bacteroides*, and three of them were markedly lower in the UN group than in the HC group (Figure 4C): *Bacteroides ovatus* (0.32 ± 1.14 and 0.90 ± 2.61% for the UN and HC groups, respectively, *P* < 0.001, FDR < 0.001), *Bacteroides uniformis* (1.70 ± 4.96 and 2.71 ± 4.37% for the UN and HC groups, respectively, *P* < 0.001, FDR < 0.001), and *Bacteroides dorei* (1.42 ± 5.51 and 4.01 ± 10.32% for the UN and HC groups, respectively, *P* < 0.001, FDR < 0.001).

### SCFAs Correlated With GM Compositions and Nutritional Indicators

**FIGURE 5:**
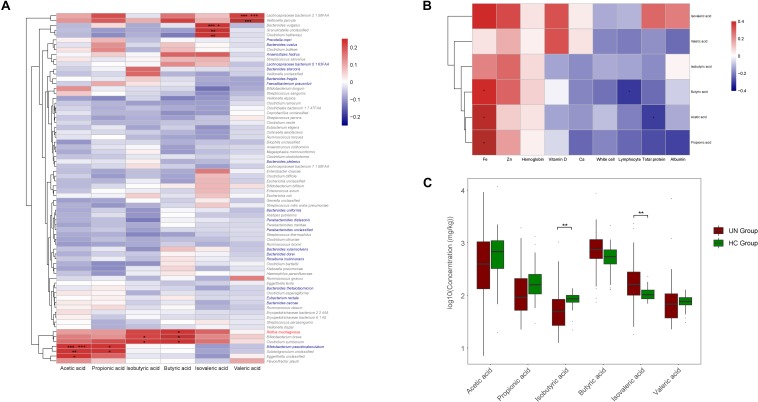
Correlations among GM, SCFAs, and nutritional indicators. **(A)** The positive and negative relationships between GM and SCFAs are exhibited with red and blue boxes, respectively. Significant relationships are indicated with “^∗^” or “+.” ^∗^*P* < 0.05, ^∗∗^*P* < 0.01, ^∗∗∗^*P* < 0.001, +FDR < 0.05, and + + + FDR < 0.001. Species labeled in red or blue were enriched in the UN or HC groups, and, for species labeled in gray, no significant differences were observed between these two groups. **(B)** The relationships between SCFAs and nutritional indicators were also explored. Red and blue boxes stand for positive and negative relationships, respectively, and ^∗^*P* < 0.05. **(C)** Comparison between the concentrations of SCFAs of the UN and HC groups. ^∗∗^*P* < 0.01.

The concentrations of SCFAs were determined for the fecal samples from 29 healthy children and 63 undernourished patients. Compared with those of the healthy children, the samples from the undernourished patients contained significantly less isobutyric acid (*P* = 0.005, FDR = 0.017) and more isovaleric acid (*P* = 0.006, FDR = 0.017) (Figure 5C). Among the six SCFAs examined, the concentrations of acetic acid and valeric acid were the highest and lowest respectively in the children’s intestines. The associations between SCFAs and GM compositions were then assessed by Spearman coefficient (Figure 5A). For the species significantly enriched in the UN group, *Rothia mucilaginosa* was positively correlated with butyric acid (*r* = 0.236, *P* = 0.038, FDR = 0.900). For the species obviously less abundant in the UN group, *Bifidobacterium pseudocatenulatum* was positively correlated with acetic acid (*r* = 0.486, *P* < 0.001, FDR < 0.001) and propionic acid (*r* = 0.275, *P* = 0.015, FDR = 0.533). Moreover, we also found positive correlations between *Lachnospiraceae bacterium* 2 1 58FAA and valeric acid (*r* = 0.652, *P* < 0.001, FDR < 0.001), *Bacteroides vulgatus* and isovaleric acid (*r* = 0.391, *P* < 0.001, FDR = 0.029), *Bifidobacterium breve* and isobutyric acid (*r* = 0.276, *P* = 0.015, FDR = 0.990), and *Bifidobacterium breve* and butyric acid (*r* = 0.257, *P* = 0.023, FDR = 0.836) (Figure 5A).

With comparisons between SCFA abundance and the nutritional indicators, we discovered that the concentrations of SCFAs were positively correlated with the levels of Fe, Zn, and hemoglobin in the blood (Figure 5B), of which Fe was positively correlated with acetic acid (*r* = 0.436, *P* = 0.029, FDR = 0.194), butyric acid (*r* = 0.422, *P* = 0.036, FDR = 0.146), and propionic acid (*r* = 0.411, *P* = 0.041, FDR = 0.185), whereas negative correlations were detected between butyric acid and lymphocyte (*r* = −0.309, *P* = 0.011, FDR = 0.103), and acetic acid and total protein (*r* = −0.303, *P* = 0.043, FDR = 0.194) (Figure 5B).

## Discussion

Childhood undernutrition is prevalent in low-income countries and is a major cause of death among children under 5 years of age (Bhutta et al., 2013). Recent studies have provided insights into the roles of the GM in nutrition absorption (Yan et al., 2016; Cani et al., 2019), and the concept of microbiota-directed intervention was proposed for its long-term amelioration on childhood undernutrition (Blanton et al., 2016a; Du Toit, 2016). However, since the GM is affected by various environmental factors (Chewapreecha, 2014; Ruiz et al., 2017; Deschasaux et al., 2018; Zmora et al., 2019), the specific GM characters of UN children should be investigated in different populations. In this study, we characterized the GM in Chinese UN patients, identified GM markers for childhood undernutrition, and explored the relationships among GM, SCFAs, and nutritional indicators.

The UN patients exhibited decreased bacterial gene and species richness when compared with healthy children, reflecting dysbiotic GM in the UN patients. However, the functions of genes or species need to be further illustrated beyond simply gene or species richness to enhance our understanding of their impacts on the host’s physical development and physiological state. A conditional pathogen that commonly colonizes the oral cavity (Maraki and Papadakis, 2015), *Rothia mucilaginosa*, was found to be enriched in the GM of the UN group. This pathogen correlates with colitis (Bajer et al., 2017) and could trigger inflammatory reactions and compete for the nutritional microenvironment based on previous reports (Subramanian et al., 2015). Since the secretion of gastric acid is reduced in UN patients (Winter et al., 2000; Gasbarrini et al., 2014), we hypothesized that the disrupted acid barrier would be beneficial for the overgrowth of *R. mucilaginosa* (Gasbarrini et al., 2014) and aggravate GM imbalance in the UN patients.

With a two-stage case–control strategy (Qin et al., 2012), 16 HC-enriched bacterial gene markers exhibited high accuracy in the discrimination of UN patients. Of the biomarkers, three were responsible for the synthesis of iron transporter. From a previous report, it was known that bacteria facilitated with iron transporters could absorb Fe^3+^ from the intestine that was unavailable to hosts and revert it to Fe^2+^ (Volkmar Braun, 2003). However, these irons can be taken of by the host through the ATP synthase in mitochondria (Qi and Han, 2018) and applied for the synthesis of hemoglobin, myoglobin, cytochrome, and enzymes. Based on these clues, we speculated that the decrease in these biomarkers would disrupt the iron homeostasis and affect body growth in the UN patients (Qi and Han, 2018). Moreover, these three gene markers came from *Bacteroides uniformis*, which was one of the species markers for the UN children. The species markers also presented with higher accuracy on the validation samples and were partially consistent with the biomarkers in Malawi UN patients, such as *Bacteroides vulgatus* (Subramanian et al., 2014). The results suggested that there are common species markers for UN patients among different populations, but the validation of the markers in different populations needs to be evaluated.

Furthermore, significantly decreased isobutyric acid and increased isovaleric acid were discovered in the UN group, and the associations between SCFAs and nutritional indicators were confirmed. An increasing number of studies have reported that isobutyric acid would promote glucose synthesis and energy harvest in the host through GPR-41 cAMP (De Vadder et al., 2014), and so the decreased isobutyric acid indicated insufficient energy collection in the UN patients. On the other hand, the accumulated isovaleric acid implied an abnormal amino acid metabolism in the patients (Loots et al., 2007), whilst high levels of isovaleric acid would cause nervous system injury (Rédei, 2008), and this phenomenon provided clues for the cognitive deficits in the UN patients. However, since the concentrations of SCFAs were positively correlated with the nutritional indicators, such as Fe, Zn, and hemoglobin, the detailed nutrition regulatory mechanisms underlying each SCFA component need to be clarified in future study.

A limitation of the current research is the validation of biomarkers in different populations. With comparison among different populations, the common pathogens related to childhood undernutrition would be discovered, and it would be helpful to develop a common treatment for UN patients worldwide. Another unresolved question is the regulatory mechanism of different SCFAs on the nutritional metabolisms in hosts. To improve our understanding of the pathogenic mechanisms of childhood undernutrition, the following issues will be addressed: (i) to validate the biomarkers in UN children with different ethnicity; (ii) to transplant the species markers into UN mouse models, and detect the impact of GM interventions on UN treatment; and (iii) to compare the physical development of UN mouse models under different SCFA interventions, and illustrate the potential roles of SCFAs in UN treatment. These studies would provide a theoretical basis for microbiota-directed therapies on childhood undernutrition.

Generally, this study described GM characteristics in Chinese UN children, identified bacterial biomarkers for childhood undernutrition, and illustrated the associations among GM, SCFAs, and nutritional indicators. These findings revealed the GM markers with high accuracy, could discriminate Chinese UN patients from controls, and supported the therapeutic potential of the GM for childhood undernutrition intervention.

## Data Availability Statement

The datasets supporting the results of this article are accessible in the NCBI Sequence Read Archive (SRA) Database under the accession number PRJNA543967.

## Ethics Statement

This study was approved by the Ethics Committee of Shenzhen Children’s Hospital under registration number 2017(010). All procedures were conducted according to the guidelines stipulated by the Ethics Committee of Shenzhen Children’s Hospital, and all clinical investigations were conducted in accordance with the principles expressed in the Declaration of Helsinki. All of the children’s parents provided written informed consent and volunteered to receive investigation on their children for scientific research.

## Author Contributions

SL, JW, and KZ managed the project. HW, WW, KY, and YY performed the fecal sampling and information collection. DL, YiL, YinhuL, and WD prepared the DNA, interpreted the analysis results, and wrote the manuscript. DL, CQ, SF, QZ, YaL, and ZY performed the bioinformatics analysis. XX, SL, and KZ optimized the graphs. SL, JW, and KZ guided the statistical analysis and polished the manuscript. All authors reviewed the manuscript.

## Conflict of Interest

The authors declare that the research was conducted in the absence of any commercial or financial relationships that could be construed as a potential conflict of interest.

## Supplementary Material

FILE S1 Physical and clinical information for all enrolled samples.

FILE S2 Concentrations of SCFAs in stools of 92 participants.

FILE S3 Statistics on the filtered data.

FILE S4 Distribution of GM in all subjects at the species level.

FILE S5 PERMANOVA ranking associations between GM and physical indicators.

FILE S6 Age-discriminatory species and their associations with age.

FILE S7 Comparison of species abundances between the UN and HC groups.

FILE S8 Comparison of gene richness between the UN and HC groups.
