# Supplementary figures and images for: Intestinal Bacteroides sp. Imbalance Associated With the Occurrence of Childhood Undernutrition in China
Source: Front Microbiol. 2019 Nov 29;10:2635. doi: 10.3389/fmicb.2019.02635 (PMC6895006; doi:10.3389/fmicb.2019.02635)

A

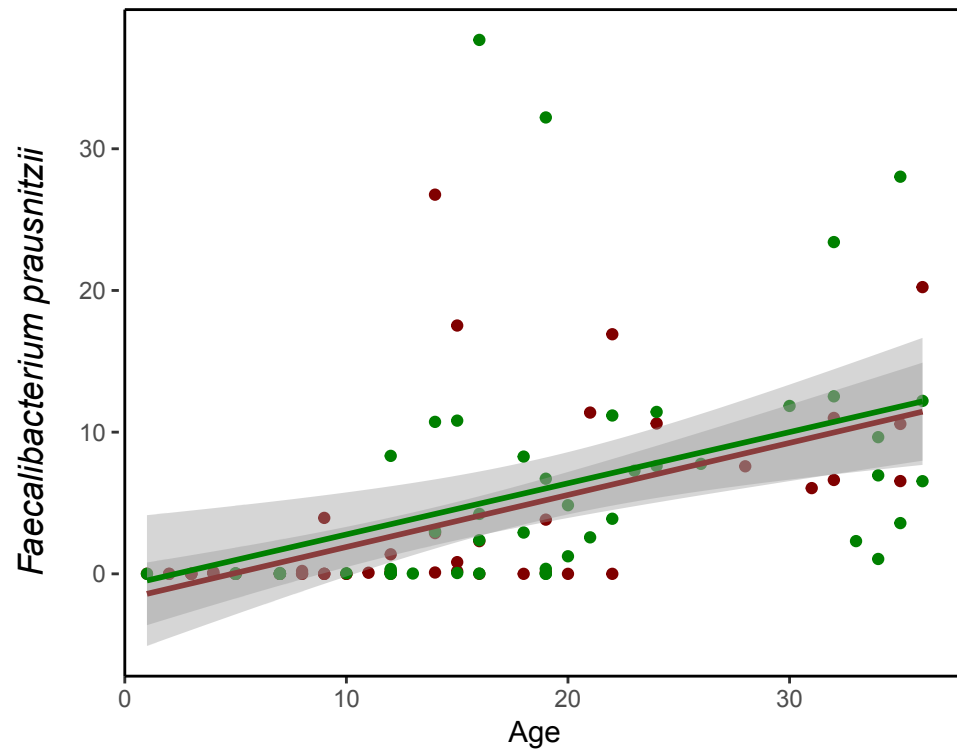

B

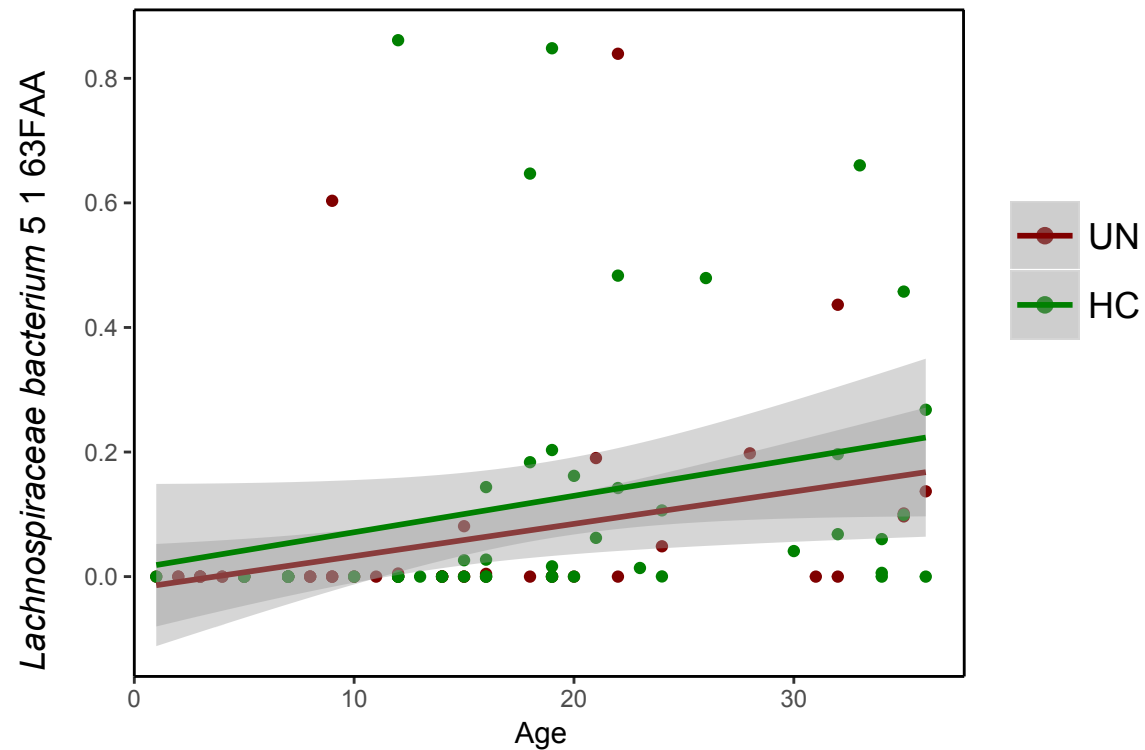

Supplement: FILE S8 — Comparison of gene richness between the UN and HC groups. [file Data_Sheet_1.PDF]
